# Primary Graft Failure after Heart Transplantation

**DOI:** 10.1155/2011/175768

**Published:** 2011-08-01

**Authors:** Arjun Iyer, Gayathri Kumarasinghe, Mark Hicks, Alasdair Watson, Ling Gao, Aoife Doyle, Anne Keogh, Eugene Kotlyar, Christopher Hayward, Kumud Dhital, Emily Granger, Paul Jansz, Roger Pye, Phillip Spratt, Peter Simon Macdonald

**Affiliations:** ^1^Heart & Lung Transplant Unit, St Vincent's Hospital, Darlinghurst, NSW 2010, Australia; ^2^Cardiac Physiology and Transplant Program, Victor Chang Cardiac Research Institute, Darlinghurst, NSW 2010, Australia

## Abstract

Primary graft failure (PGF) is a devastating complication that occurs in the immediate postoperative period following heart transplantation. It manifests as severe ventricular dysfunction of the donor graft and carries significant mortality and morbidity. In the last decade, advances in pharmacological treatment and mechanical circulatory support have improved the outlook for heart transplant recipients who develop this complication. Despite these advances in treatment, PGF is still the leading cause of death in the first 30 days after transplantation. In today's climate of significant organ shortages and growing waiting lists, transplant units worldwide have increasingly utilised “marginal donors” to try and bridge the gap between “supply and demand.” One of the costs of this strategy has been an increased incidence of PGF. As the threat of PGF increases, the challenges of predicting and preventing its occurrence, as well as the identification of more effective treatment modalities, are vital areas of active research and development.

## 1. Introduction

Heart transplantation is an effective method of treatment for end-stage heart failure, with more than 5,000 transplants being conducted each year in over 300 countries [1]. The survival rate after heart transplantation has improved steadily over the last two decades with virtually all of the improvement being in survival during the first few months [1]. Despite this improvement in early post-transplant survival, there is little if any evidence that deaths due to primary graft failure (PGF) have decreased over this period. In a large retrospective study of 7,259 heart transplant recipients during the decade from 1990 to 2000, Young and colleagues reported that the one month mortality after heart transplantation was 6.9% with 43% of these deaths due to PGF [2]. This compares with the most recent audit of the International Society of Heart and Lung Transplantation Registry which reported a one month mortality after transplantation of 8% with 39% of these deaths resulting from PGF [1]. It is clear from these data that PGF continues to be the single most common cause of death within the first month after heart transplantation [1]. In addition, the high morbidity associated with PGF and its treatment is likely to be a major contributor to deaths that are attributed to other causes such as infection and rejection over subsequent months. 

## 2. Incidence

The reported incidence of PGF after heart transplantation varies widely between studies with estimates ranging between 2.3 and 26% [3–11]. Most of the variability can be attributed to inconsistent definitions of PGF used by different authors. In a large retrospective review of the UNOS Registry, Russo and colleagues defined PGF as death or retransplantation within the first 90 days of transplantation and reported an incidence of only 2.5% [5]; however, as argued by others, the use of such a definition based on “hard endpoints” is likely to underestimate the true incidence of the clinical syndrome as it only detects those with the worst clinical outcomes [12, 13]. In contrast, when PGF has been defined as the need for high-dose inotropes or mechanical assist devices in the immediate post-transplant period, most investigators have reported incidence rates of 10–20% or higher [3, 4, 7–9].

The changing demographics of donors and recipients observed in cardiac transplantation over the last two decades appear to be contributing to an increase in the incidence of PGF [9, 11, 14]. Transplant centres face significant donor shortages and growing waiting lists. This is no more evident than in Australia, where the combination of a relatively small population and low organ donation rate has resulted in increased utilisation of hearts from older “marginal” donors [11, 14, 15] and suboptimal organs from younger donors. In addition to this, greater procurement distances to retrieve donor hearts in Australia contribute to prolonged ischaemic times. The combination of these two factors, advanced donor age and prolonged ischaemic time, markedly increases the risk of PGF and death after heart transplantation [5, 16].

## 3. Definition and Diagnostic Criteria

PGF is a syndrome in which the transplanted heart fails to meet the circulatory requirements of the recipient in the immediate post-transplant period as a consequence of either single or biventricular dysfunction. It is manifested as hypotension and low cardiac output in the presence of adequate filling pressures [17]. In most instances, it is likely to result from a multifactorial process with contributing elements from the donor, recipient, and the transplant process. 

A universally accepted clinical definition for PGF has been lacking and is urgently needed. Several authors have suggested minimal diagnostic criteria [7, 9], which are summarised in Table 1. The primary diagnostic criterion for PGF is evidence of ventricular dysfunction which may involve the left, right, or both ventricles occurring within the first 24 hours of heart transplantation. The major clinical manifestation of this dysfunction is severe haemodynamic instability with cardiogenic shock. A diagnosis of PGF should only be made when other causes of acute graft failure such as cardiac tamponade and hyperacute rejection have been excluded.

 The severity of PGF can be graded according to the level of support needed to restore haemodynamic stability. In less severe cases, intravenous inotropic support with two or more agents may be sufficient to achieve this, whereas in more severe cases mechanical circulatory assistance (including intra-aortic balloon pump, extracorporeal membrane oxygenator (ECMO), or any ventricular assist device) is required. A three-level grading system based on the severity of primary graft dysfunction has been developed for lung transplantation and shown to be strongly predictive of one-month mortality [18, 19]. It seems likely therefore that the severity of cardiac PGF has an equally significant prognostic value after transplantation. In view of this, a standardised clinical definition of PGF incorporating a severity grading system is urgently needed.

## 4. Aetiology and Pathogenesis

Acute ischaemia-reperfusion injury with myocardial stunning has been postulated as a predominant factor in the development of PGF. The donor heart is subjected to a series of insults during the transplant process including brain death and its sequelae, hypothermic storage, warm ischaemia, and finally reperfusion. Donor hearts vary in their ability to withstand these insults. It is clear, for example, that the hearts from older donors have an increased susceptibility to PGF [5, 16] which may be explained by the observation that aged myocytes have a reduced ability to withstand ischaemia-reperfusion injury [20].

Brain death in the donor is associated with a series of events that result in impaired myocardial contractility. These events include the rapid release of catecholamines immediately after brain death contributing to myocardial ischaemia, calcium overload, calpain activation, and changes in the calcium sensitivity of contractile proteins [21, 22]. The surge in endogenous catecholamine release immediately after brain death followed by the administration of exogenous catecholamines during donor resuscitation may contribute to desensitization of the myocardial beta-receptor signal transduction system after brain death and to the activation of multiple proinflammatory mediators [23–26]. In addition, decreased serum levels of various hormones including triiodothyronine, cortisol (after a transient increase), and insulin have been reported and likely contribute to the depression of myocardial contractility [27]. 

Most donor hearts are stored in a cold preservation solution and transported on ice. Hypothermic storage slows but does not completely arrest cellular metabolism. Consequently, progressive ischaemic injury is an inevitable consequence of prolonged static storage. In addition, loss of normal aerobic metabolism paralyses the transmembrane Na+/K+ ATPase pump leading to cellular swelling and the switch to anaerobic metabolism during cold storage results in a rapid decline in high-energy phosphates and the development of lactic acidosis [28]. Finally, reperfusion injury results in further calcium overload and oxidative stress both of which can contribute to the mechanism of stunning [21, 28]. Thus, at every stage of the transplant process, the heart is exposed to cellular stresses that may adversely impact on myocardial function and ultimately lead to the syndrome of PGF.

Primary graft failure may also occur in circumstances where the donor heart has not been subjected to substantial ischaemia-reperfusion injury. Under these circumstances, recipient factors are the principal cause of PGF. There are two clinical scenarios where this is likely to occur. The first is the presence of a fixed high pulmonary vascular resistance in the recipient [29–31]. In this circumstance, the right ventricle of the donor heart is unable to overcome the afterload imposed by the elevated pulmonary vascular resistance, and selective or predominant right ventricular failure ensues. In one series of 911 patients, 28 of 130 deaths were due to acute graft failure with 43% of this early mortality (12 of 28 patients), attributed to severe preoperative pulmonary hypertension causing right-sided circulatory failure, low cardiac output and eventually biventricular failure [32]. The second scenario is when the recipient is critically ill on ventilatory and/or acute mechanical circulatory support often with evidence of multisystem failure and sepsis [2, 3, 5]. In this circumstance, the “hostile environment” of the recipient results in PGF. The pathophysiology of PGF in this setting is poorly understood but probably involves the concerted action of multiple proinflammatory cytokines on the transplanted heart. 

In most instances, it is likely that the combination of donor, procedural and recipient factors leads to the syndrome of PGF. For example, an older donor heart that has been subjected to a prolonged ischaemic time may fail in a recipient with an elevated pulmonary vascular resistance whereas a younger donor heart may not. On the other hand, the same older donor heart may function adequately in a haemodynamically stable recipient with low pulmonary vascular resistance. Hence, matching donors to recipients with regard to risk factors for PGF are critical to minimising the risk of this life-threatening complication.

## 5. Risk Factors for PGF

Given the significant contribution of PGF to early mortality after cardiac transplantation, identification of predictive factors is important. Multiple risk factors for PGF have been identified by different authors. They can be divided into those that are donor related, those that are recipient related and those related to the transplant procedure. 

As shown in Table 2, multiple donor and recipient factors have been associated with an increased risk of PGF. Principal among these are increasing donor and recipient age, both of which have also been identified as major risk factors for one-year mortality after transplantation [1]. The review of the Australian & New Zealand Cardiothoracic Transplant Registry reveals that there has been a steady rise in mean donor and recipient age over the last 2 decades [15] with the mean donor age exceeding 40 years of age for the first time in 2010 (personal communication with Mr. Ross Pettersson). 

Another potent risk factor for PGF identified in multiple studies is donor heart ischaemic time, referring to the period from the arrest of the donor heart to time of graft reperfusion in the recipient. It is apparent from the ISHLT Registry that one-year mortality risk after heart transplantation increases steadily with every minute of ischaemic time in excess of 3 hours [1]. Marasco et al. estimated that the risk of PGF increased by 43% for every hour of extra ischaemic time beyond 4 hours [7]. As with donor age, there has been a significant increase in donor heart ischaemic time for heart transplants performed in Australia and New Zealand from a mean of less than 3 hours prior to 1990 to a mean in excess of 4 hours for most of the last decade [15]. In our own recently reported experience of ECMO support for PGF, donor heart ischaemic times of 5 hours or longer were associated with a fivefold increase in the risk of PGF [11]. 

These data indicate that the current techniques used to preserve the donor heart during procurement and transport have limited efficacy. Unfortunately, prolonged ischaemic times in heart transplantation are sometimes logistically unavoidable. There is a clear need to develop more effective preservation strategies—either by bolstering the cardioprotective efficacy of the storage solution or through use of oxygenated ex vivo perfusion systems. Counter-intuitively, Russo et al. reported an increased risk of PGF with ischaemic time of less than 1 hour, citing the potential limited cooling period being insufficient to achieve the benefits of cellular protection with global hypothermia [5]. 

Several authors have reported that donor heart dysfunction as evidenced by a low left ventricular ejection fraction on echocardiography, unstable donor haemodynamics, or the need for high doses of catecholamines is a potent risk factor for PGF [2, 3, 6, 11]. Historically, donor hearts that displayed these characteristics would have been regarded as unsuitable for transplantation; however, increased demand for transplantation has led to many Transplant Units including our own making use of these “marginal” hearts [3, 6, 11, 14]. The expectation is that the myocardial dysfunction evident in the donor is a result of stunning and is recoverable over time despite the current lack of a useful clinical measure that can reliably distinguish reversible from irreversible myocardial dysfunction in the brain dead donor. Of all the clinical information available regarding a potential heart donor, a young donor age (<30 years) and the absence of any known history of heart disease are probably the two pieces of clinical information that our group most relies on when deciding to use a donor heart with overt myocardial dysfunction prior to procurement [11]. 

Donor-recipient size mismatch has also been identified as a significant contributing factor in the development of PGF. In one study, the combination of a donor-recipient weight ratio of less than 0.8 with pulmonary hypertension in the recipient (>4 wood units) was associated with PGF [5]. Several studies have found that the transplantation of a female donor heart into a male recipient was associated with increased PGF, with size mismatch being the likely connection. A possible link to immunological processes and increased rejection episodes have also been described [2, 35]. 

The concurrent donation of other organs may also have a role in PGF, specifically the donation of lungs [5]. The proposed aetiologies include additional flush volume that may contribute to RV distension and dysfunction, and release of pulmonary vascular cytokines at time of arrest which can result in ventricular dysfunction [5]. The association of PGF in multiple organs retrieved from the same multi-organ donor has also been reported, highlighting the potential for significant donor influences in the development of PGF [8]. This also allows predictability of PGF through monitoring of other organs transplanted from that specific donor [8]. 

The presence of ventilator or ECMO support in the recipient prior to and at the time of transplantation has been shown to be a significant risk factor for PGF [2, 5]. These patients are usually critically ill with evidence of multi-organ dysfunction and often sepsis. Conversion of these patients to long-term mechanical support with a left ventricular assist device or total artificial heart is associated with significant mortality [38], but if successful enables resolution of any acute multi-organ dysfunction with subsequently safer transplantation when the patient's condition has stabilised. Although a trend to increased PGF has been reported in patients who are bridged to transplantation with long-term implanted VADs [5], post-transplant survival of these patients does not appear to be compromised [15, 39]. 

Risk factors do not act in isolation, and it is likely that the interaction between donor, recipient, and procedural factors is a major determinant of the risk of PGF. A clear example of this is the interaction between donor age and ischaemic time reported by Russo et al. [16]. In that study, there was no detectable adverse effect of ischaemic time on survival after heart transplantation when the donor was less than 20 years of age. In contrast, when the donor age increased above 20 years, a prolonged ischaemic time had a significant negative impact on survival [17]. This effect became even more marked when the donor age exceeded 35 years. The association of increasing donor age with PGF is likely related to the decreased ability of the aging heart to tolerate ischaemic insults as well as the increased incidence of intrinsic cardiac pathology with age [20]. 

### 5.1. PGF Predictive Tool

Given that multiple factors in the donor, the transplantation process and the recipient contribute to the risk of PGF, the development of a predictive tool, and scoring system that combines known risk factors has been reported [9, 40]. Suggested variables have included donor and recipient age, donor inotropic dependence, recipient right atrial pressure, and ischaemic time [9]. With further understanding of the aetiology of PGF, as well as identification and confirmation of risk factors, an accurate predictive scoring tool is imminent in the near future. The utility of any predictive tool remains to be determined, but it does serve to emphasise the importance of careful donor-recipient matching in the prevention of this life-threatening complication.

## 6. Management

The treatment of PGF remains extremely challenging—a substantial 30-day mortality rate is seen despite intensive pharmacological as well as mechanical circulatory support (IABP, ECMO, VAD) used in this critical period [1, 2]. In milder cases of primary allograft dysfunction, high-dose inotropic agents may be sufficient to restore myocardial contractility and haemodynamic stability. A variety of inotropic agents have been used to treat PGF include catecholamines, phosphodiesterase inhibitors, and more recently levosimendan [41–43]. 

With more severe cases of graft failure, mechanical circulatory support with intra-aortic counterpulsation or VA extracorporeal mechanical support (ECMO) may be needed to maintain haemodynamic support and perfusion of vital organs. In our institution, the decision to institute ECMO has been made early, that is, in the operating room when there has been difficulty with separating from cardiopulmonary bypass despite a trial of inotropic/vasopressor support [11]. We believe that early institution of ECMO not only allows the heart more time to recover from the multiple stresses to which it has been exposed but also prevents development of multisystem organ failure which would otherwise occur if there is a period of uncorrected cardiogenic shock. Recent advances in ECMO circuit design have resulted in a significantly improved survival rates and fewer complications compared with practice not longer than a decade ago, when paracorporeal ventricular assist devices were used for left ventricular support and centrifugal pumps for right ventricle support [7, 11, 44, 45]. 

Heart transplant recipients with PGF remain supported on ECMO until graft function improves. In our experience, this has generally been within 72 hours; however, heart recovery has been observed as early as 1 day and as late as 7 days after transplant [11]. Assessment of the timing of cardiac recovery is usually judged by daily bedside echocardiography with brief reduction in ECMO flow during echocardiographic examination. The majority of the patients in our series have had peripheral femoral venous and arterial cannulas placed for ECMO support, and in most cases, it has been possible to remove these cannulae in the intensive care unit without the need to return to the operating theatre. 

In cases with pre-existing recipient pulmonary hypertension, PGF is usually manifested as right ventricular dysfunction in the immediate post-transplant period. Treatment includes administration of specific pulmonary vasodilators such as inhaled nitric oxide to lower pulmonary vascular resistance [46] however, mechanical circulatory support may be needed [45]. Long-term administration of selective pulmonary vasodilators (prostacyclin, sildenafil) or in some cases implantation of left ventricular assist devices in potential heart transplant recipient with fixed pulmonary hypertension has been reported to produce sustained lowering of pulmonary vascular resistance allowing orthotopic heart transplantation to be performed without any increase in perioperative graft failure or mortality [47, 48]. 

## 7. Prognosis

PGF is the leading cause of death in the first month after heart transplantation. Although registry studies indicate that the number of early deaths due to PGF has not changed over the last two decades [1, 2], this is in the setting of an increasing incidence of PGF reported in the literature [3, 4, 11, 14]. This suggests that the prognosis for patients diagnosed with PGF is improving, most likely as a result of the improved efficacy and safety of pharmacological and ECMO support in these critically ill patients [3, 11, 14]. In our own experience of 17 patients supported on ECMO for PGF, one month survival was 82% [11].

The impact of PGF beyond the first month after transplantation is less clear, but also likely to be significant. Severe ischaemia-reperfusion injury has been shown experimentally to upregulate multiple proinflammatory mediators which may prime the graft for acute rejection [25, 26] and also predispose the graft to allograft vasculopathy [49], both of which could contribute to graft failure at later time points.

## 8. Prevention and Areas for Future Improvement

Given the cumulative impact of the multiple risk factors that contribute to the development of PGF, careful matching between donor and recipient is critical to minimising the risk of PGF. Unfortunately, the logistics of transplantation sometimes dictate that unfavourable risk factor interactions cannot be avoided. While some risk factors (e.g., donor and recipient age) are not modifiable, other risk factors (e.g., donor heart ischaemia-reperfusion injury sustained following brain death or during organ procurement and preservation) may be amenable to therapeutic intervention. 

The period between brain death and heart retrieval is one in which heart function can deteriorate rapidly. Optimal management of the brain dead donor during this period remains a contentious issue. More than 90% of brain dead donors receive one or more inotropic or vasopressor infusions most commonly noradrenaline [50]. While low-dose infusions of catecholamines appear to be safe, high-dose infusions increase the risk of PGF and should be avoided [3, 6, 34]. There has been a longstanding interest in the administration of pituitary-dependent hormones in the optimisation of donor organ quality after brain death. Vasopressin is an effective alternative to noradrenaline for maintaining blood pressure, and its use may prevent the need for escalating doses of noradrenaline [51, 52]. On the other hand, the value of thyroid hormone and corticosteroids in this setting is still controversial. While large-scale retrospective analyses support a role for these drugs [53, 54], prospective randomised controlled trials to date have failed to demonstrate any improvement in cardiac function or outcome after transplantation [55, 56].

The period of heart storage and transport is the second period that offers an opportunity to intervene. Currently most hearts are stored and transported in cold cardioplegic/preservation solutions. The many commercial and in-house cardioplegic/preservation solutions in routine clinical use not only emphasise the complexity of the molecular and cellular mechanisms that underlie ischaemia-reperfusion injury but also the lack of consensus as to the optimal strategy for organ preservation [57]. Cardioplegic/storage solutions such as St Thomas' Solution No. 2 (Plegisol), Bretschneider (Custodiol), and Celsior, have been in widespread clinical use since the early 1990's [58–60] and appear to provide adequate protection of “standard criteria” of donor hearts subjected to ischaemic times of less than 4 hours [1]. The cardioprotective capacities of such formulations may be suboptimal for the increasing numbers of “marginal” donor hearts seen in current clinical practice, particularly those subjected to prolonged ischaemic times. 

Elucidation of the mechanisms of ischaemia-reperfusion injury over this same period of time has suggested novel strategies to enhance the cardioprotective capacities of existing preservation solutions. The search for an over-arching protective strategy against cardiac reperfusion injury has been advanced by the realisation that ischemic pre- and postconditioning as well as a number of pharmacological agents that mimic these physiological strategies can activate prosurvival signalling pathways such as PI3K/Akt, ERK 1/2 and STAT3 at reperfusion (for review see Hausenloy et al., [66]). Consistent with this mechanism, we have recently demonstrated that rat hearts arrested and stored for 6 or 10 hours in Celsior solution supplemented with the conditioning agents glyceryl trinitrate (GTN), a nitric oxide donor, and cariporide, a sodium hydrogen exchange inhibitor significantly improved poststorage cardiac function that could be abolished by inhibition of the mitochondrial K_ATP_ channel, a key target of prosurvival signalling pathways [67]. These findings have recently been further verified in a translational porcine orthotopic heart transplant model incorporating donor brain death. Here, donor hearts arrested 6 hours after brain death and stored in Celsior supplemented with GTN and cariporide could be successfully weaned from cardiopulmonary bypass after 14-hour hypothermic storage [68]. In addition, we have demonstrated that appropriate pharmacological supplementation of the arresting and storage solution can activate survival signalling after reperfusion in a model of a normal donor heart exposed to storage times that would class them as “marginal” (6 hr storage) or unsuitable for transplant (10 hour storage) (Table 3).

An alternative to cold static storage is ex vivo perfusion. There is limited experience with this approach in heart transplantation [69]; however, a recent large randomised controlled trial in deceased kidney transplantation revealed a significant reduction in primary graft dysfunction and improved graft survival at one year after transplant in machine-preserved kidneys [70]. These benefits were particularly marked in kidneys obtained from marginal donors [71]. 

In summary, the increasing reliance on “marginal” donors to meet the ever-increasing demand for heart transplantation means that PGF is likely to remain a frequent complication. Although there have been significant improvements in the treatment of established PGF, it still carries a high morbidity and mortality. While it is possible that some cases of PGF may be prevented by careful matching of donors and recipients, complete prevention of PGF will require the development of more effective donor management and donor heart preservation strategies. These remain high-priority areas for ongoing basic and clinical research.

## Figures and Tables

**Table 1:** Suggested diagnostic criteria for primary graft failure [7, 9].

Presence of	Evidenced by
Ventricular systolic dysfunction—left, right, or biventricular dysfunction	Echocardiographic evidence of dysfunction

Cardiogenic shock lasting more than one hour	Low systolic blood pressure < 90 mmHg and/or low cardiac output—<2 L/minute/m^2^ Despite adequate intracardiac filling pressures—CVP > 15 mmHg and/or PAWP > 20 mmHg

Circulatory support	Use of ≥2 inotropic agents/vasopressors including high-dose epinephrine or norepinephrine and/or use of a mechanical assist device—IABP, ECMO, VAD

Appropriate time frame	Onset < 24 hours after transplantation

Exclusion of secondary causes of PGF	For example, cardiac tamponade and hyperacute rejection

**Table 2:** Risk factors for primary graft failure.

Donor factors	Recipient factors	Procedural factors
Age [2, 5, 7, 9, 16, 33]	Age [3, 33]	Ischaemic time [2, 3, 7, 9, 11, 33]
Cardiac dysfunction on echo [2, 3, 11]	Ventilator support [2]	Donor recipient weight mismatching [2]
High-dose inotropic support [3, 6, 34]	Intravenous inotropic support [9], Mechanical support [3, 5]	Female donor to male recipient [2, 5, 35]
Cause of brain death [3, 36]	Pulmonary hypertension [17, 29–31]	Concomitant lung retrieval [5]
Primary graft dysfunction of other organs [8]	Overweight [37], Diabetes mellitus [9]	

**Table 3:** Pharmacological activation of prosurvival kinases in a model of donor heart preservation.

Agent (s)^1^	Storage time (h)	Poststorage CO recov^2^	Prosurvival kinase phosphorylation^3^			Other salient findings	Ref.
			Akt	ERK	STAT3		
GTN (0.1 mg/mL)	6	2.5	0	+	nd^4^	↓ cleaved Casp 3	[61]
Carip (10 *μ*M)	6	3.5	0	++	nd^4^	↓ cleaved Casp 3	[61]
INO 1153 (1 *μ*M)	6	2.5	+++	+	nd^4^	Recovery of function abolished by Akt inhib	[62]
Zonip (1 *μ*M)	6	14	0	+++	+++	Zonip abolished LDH release; ↓ cleaved Casp 3; Inhib of STAT3 phos abolished recovery of f'n.	[63]
Neureg (14 nM)	6	13	++	+++	+++	Recovery of function abolished by Akt inhib	[64]
EPO (5 units/mL)	6	16	0	0	+++	Inhib of STAT3 phos abolished recovery of f'n.	[65]
Neureg + GTN + Carip	10	13	++	0	+	Triple supplement ↓ contraction band necrosis	[64]

^1^Agent(s) added to Celsior arresting and storage solution. Abbreviations/drug classes are as follows: GTN—glyceryl trinitrate (nitric oxide donor); Carip—cariporide, Zonip—zoniporide, (both sodium/hydrogen exchange inhibitors); INO 1153—poly(ADPribose) polymerase inhibitor; Neureg—recombinant human Neuregulin-1 peptide; EPO—erythropoietin. ^2^Recovery of cardiac output expressed as fold increase over Celsior-stored hearts (*P* ≤ 0.05); ^3^increase in survival kinase phosphorylation over Celsior-stored hearts; +++: intense; ++: moderate; +: weak; ^4^nd-not determined;
